# Rb1, the Primary Active Ingredient in *Panax ginseng* C.A. Meyer, Exerts Antidepressant-Like Effects *via* the BDNF–Trkb–CREB Pathway

**DOI:** 10.3389/fphar.2019.01034

**Published:** 2019-09-13

**Authors:** Guoli Wang, Cong Lei, Ya Tian, Yingping Wang, Lianxue Zhang, Ronghua Zhang

**Affiliations:** ^1^College of Pharmacy, Jinan University, Guangzhou, China; ^2^College of Chinese Medicinal Materials, Jilin Agricultural University, Changchun, China

**Keywords:** ginseng, fibrous roots, flower buds, main roots, ginsenoside Rb1, antidepressive effects, chronic unpredictable mild stress, BDNF–TrkB–CREB signaling

## Abstract

*Panax ginseng* C.A. Meyer (Araliaceae), a popular tonic and dietetic herbal medicine, has been traditionally prescribed in China and other countries to treat affective disorders. The medicinal parts of ginseng, the roots and flower buds, have become increasingly popular as dietary supplements due to the current holistic healthcare trend. We have investigated for the first time the antidepressive actions of the different medicinal parts, namely, the main roots, fibrous roots, and flower buds (in water extract and powder), of garden-cultivated ginseng through behavioral and drug-induced tests in mice. The water extracts, but not the powders of ginseng fibrous roots, flower buds, and main roots (1.5 g of crude drug per kilogram, p.o.), significantly reduced the immobility time in the forced swim test (FST) and tail suspension test (TST); moreover, the water extracts enhanced the 5-hydroxytryptophan (5-HTP)-induced head-twitch response and antagonized the action of reserpine in the mouse. We then explored the antidepressive mechanism of action of the ginsenoside Rb1 (Rb1) related to the brain-derived neurotrophic factor (BDNF) and its downstream proteins in mice exposed to chronic unpredictable mild stress (CUMS). Treatment with Rb1 (20 mg/kg, p.o.) for 21 days significantly attenuated the CUMS-induced decrease in the activities of BDNF, tropomyosin-related kinase B (TrkB), protein kinase B (AKT), extracellular regulatory protein kinase (ERK), and cyclic adenosine monophosphate (cAMP) response element binding protein (CREB) in the mouse hippocampal CA3 region and prefrontal cortex (PFC). Interestingly, treatment with the novel TrkB antagonist ANA-12 (0.5 mg/kg, i.p.) did not alter the level of BDNF but significantly blocked the antidepressive effects of Rb1 on proteins downstream of BDNF in CUMS-treated mice. These results suggest that BDNF–TrkB–CREB signaling may be involved in the antidepressive mechanism of the action of Rb1.

## Introduction

Depression is the leading cause of disability, with a prevalence greater than 20% in the global population (Lavanya et al., 2017). Currently, depression is underdiagnosed and undertreated in primary healthcare systems and results in significant emotional and economic burdens for patients and their families. Although the precise neurobiology of depression is unknown, several lines of evidence indicate that neurotrophic factors are involved in depressive symptoms (Levy et al., 2018; Alves and Rocha, 2018).

Brain-derived neurotrophic factor (BDNF) and its receptor, tropomyosin-related kinase B (TrkB), play key roles in the pathogenesis of depression and serve many critical functions in neuronal maturation, synapse formation, and synaptic plasticity (Park and Poo, 2013). Cyclic adenosine monophosphate (cAMP) response element binding protein (CREB) is an important transcription factor in the brain, controlling the biosynthesis of many prosurvival proteins, including BDNF (Luo et al., 2017). Binding of BDNF to the TrkB receptor is known to promote two key downstream signaling pathways, namely, the mitogen-activated protein kinase (MAPK)/extracellular regulatory protein kinase (ERK) and phosphatidylinositol 3-kinase (PI3K)/protein kinase B (PKB/AKT) signaling pathways that induce CREB phosphorylation and activation (Shaywitz and Greenberg, 1999; Lim et al., 2008). Clinical studies have confirmed that BDNF is a key transducer of antidepressive effects. Deficiency of BDNF–TrkB–CREB function induces susceptibility to depression in rodents, while administration of BDNF–TrkB–CREB elicits antidepressant-like effects in animal models of depression (Hoshaw et al., 2005; Advani et al., 2009). These findings indicate that BDNF–TrkB–CREB signaling has potential as a therapeutic target in treating depression (Hashimoto et al., 2004; Hashimoto, 2010; Zhang et al., 2016).

Although a variety of synthetic chemical antidepressants are currently being evaluated in clinical trials, they have therapeutic limitations related to cardiotoxicity, suicidal tendencies, sexual dysfunction, and sleep disorders (Amiri et al., 2016; Metzger et al., 2017; Polychroniou et al., 2018; Clayton et al., 2018). Chinese herbal remedies with less harmful side effects and, therefore, higher levels of safety, have been developed as both conventional and alternative drugs (Cassani et al., 2015).

**Figure 1:**
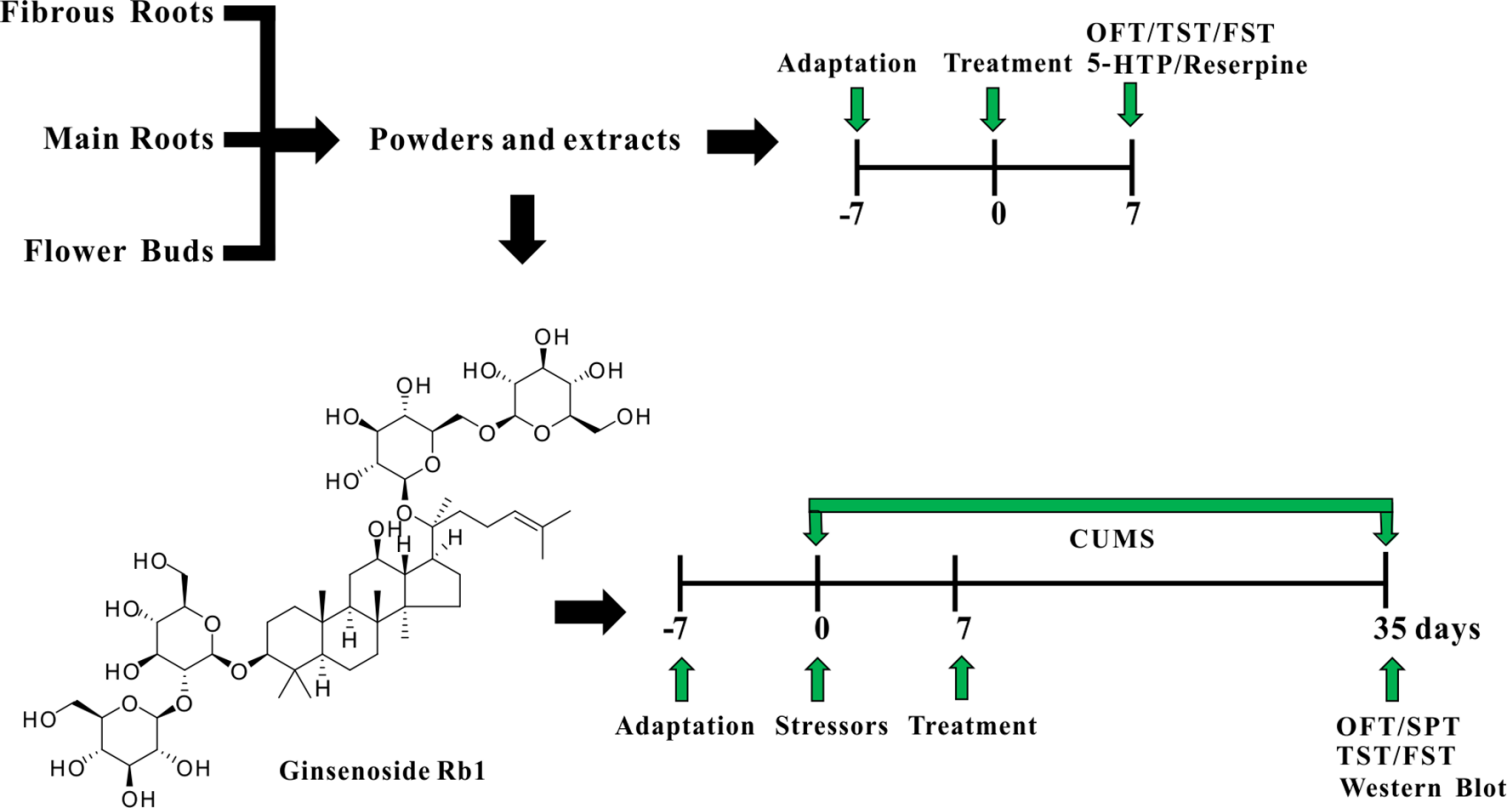
A detailed illustration of the current experiment.

*Panax ginseng*, known in China as the “King of Chinese Medicines,” is a prominent functional food traditionally prescribed to treat affective disorders in China and other countries (Seok Rye et al., 1996; Lee et al., 2011). The medicinal parts of ginseng (roots and flower buds) are increasingly popular as dietary supplements due to the current trend towards a holistic approach to healthcare. Although numerous extraction methods exist in the typical home, including supercritical fluid, ultrasonic wave, microwave, and heat reflux (Dai and Orsat, 2010; Wang et al., 2010; Ajavakom et al., 2012; Johner et al., 2018; Zhang et al., 2018) traditional water extraction and powdering are the most frequently used methods to obtain the medicinal benefits of ginseng *via* ingestion (Nabavi et al., 2018; Taban and Saharkhiz, 2015). The main effective constituents of ginseng are the ginsenosides, of which Rb1, a protopanaxadiol (PPD)-type ginsenoside with a dammarane-type triterpenoid as an aglycone, is the main bioactive component (Quan et al., 2011). Our previous studies confirmed that Rb1 elicits a novel, antidepressant-like effect by regulating monoamine and amino acid neurotransmitter levels in the hippocampal CA3 region and prefrontal cortex (PFC) (Wang et al., 2017; Wang et al., 2018); however, how Rb1 regulates these neurotransmitters, and whether the mechanism involves neurotrophic molecules is unknown. In the present study, therefore, we investigated for the first time the antidepressive effects of the common medicinal parts of garden ginseng, namely, the main roots, fibrous roots, and flower buds (in water extract and powder), through behavioral and drug-induced tests in mice. We then explored the mechanism of antidepressive action of the Rb1 *via* the BDNF–TrkB–CREB signaling pathway in the hippocampus and PFC of mice exposed to chronic unpredictable mild stress (CUMS). A detailed illustration of the current experiment is shown in **Figure 1**.

## Materials and Methods

### Experimental Animals

Male mice (22–24 g) from the Institute of Cancer Research (ICR) were procured from Yi-Si Laboratory Animal Technology Co., Ltd (Changchun, China). The animals were housed in groups under standard laboratory conditions (room temperature 25 ± 1°C, 12-h light/dark cycle, humidity set to 40–70%) and were provided with water and food *ad libitum*. The mice were allowed to habituate to the novel environment for 1 week and were then randomly divided into groups of 10 mice per cage. The animals were maintained in accordance with the institutional guidelines of the National Institutes of Health, and all tests were approved by the Animal Care Committee of Jilin Agricultural University (Permit No. ECLAJLAU-17005).

### Drugs and Reagents

Dry fibrous roots, main roots, and flower buds of 6-year-old garden ginseng (Kang-Mei No. 1) were provided by our laboratory and identified by Professor Lian-Xue Zhang (Department of Chinese Medicinal Materials, Jilin Agricultural University). A voucher specimen (Jidengyao 2012002) was deposited in the Engineering Research Center of Ginseng, Jilin Agricultural University. Half of each material (fibrous roots, main roots, and flower buds) were pulverized into a fine powder (120-mesh sieve). The other half was made into a liquid extract, where 100 g each of dried fibrous roots, main roots, and flower buds was extensively extracted with 1,000, 800, and 600 ml of distilled water for 2, 1, and 1 h, respectively. The filtrate was combined and then freeze-dried to obtain the dry extract of fibrous roots (yield 40.1%), main roots (yield 36.2%), and flower buds (yield 44.5%). The freeze-dried samples were stored at −20°C until use.

Twenty ginsenosides (Rg5, F2, Rb1, Rb2, Rb3, Rc, Compound K, Rd, Rg3, Rh2, PPD, Rf, Rg1, Rg2, Re, F1, Rk3, Rh1, Rh4, and PPT) in the powder of fibrous roots (PFRs), flower buds (PFBs), and main roots (PMRs) and the water extract of fibrous roots (EFRs), flower buds (EFBs), and main roots (EMRs) (%) were determined by high-performance liquid chromatography (HPLC) using an Agilent 1260 Infinity LC (Agilent Technologies, USA). The separation was achieved on an Eclipse XDB-C18 column (250 mm × 4.6 mm, 5 μm, USA). The mobile phase consisted of acetonitrile (Fisher Company, USA) (solvent A) and water (solvent B) with the following gradient program: 0–40 min, 18–21% A; 40–42 min, 21–26% A; 42–46 min, 26–32% A; 46–66 min, 32–33.8% A; 66–71 min, 33.8–38% A; 71–77.7 min, 38–49.08% A; 77.7–78 min, 49.08–49.1% A; 78–82 min, 49.1% A; 82–83 min, 49.1–50.6% A; 83–88 min, 50.6–59.6% A; 88–89.8 min, 59.6–64.96% A; 89.8–92 min, 64.96–65% A; 92–97 min, 65% A; 97–102 min, 65–85% A; 102–109 min, 85% A; and 109–111 min, 85–18% A. The flow rate was 1 ml/min (10-μl injection volume) with absorbance detection at 203 nm. The HPLC results of the different ginseng medicinal parts are shown in **Figure S1** and **Table S1**. Rb1 (C54H92O23, molecular weight 1,109.31, **Figure 1**) was separated by preparative liquid chromatography to a purity of >98%; its ^13^C NMR spectra and data are shown in **Figure S2** and **Table S2**, respectively, and agreed well with those reported in our previous studies (Ruan et al., 2010; Wang et al., 2016).

Fluoxetine and 5-HTP were obtained from Melone Pharma Co. Ltd (Dalian, China). Reserpine was purchased from Aladdin Co. Ltd (Shanghai, China).

### Treatment Schedules

For examination of the antidepressant activity of the different ginseng medicinal parts, mice were orally administered a control (Con, 0.5% carboxymethyl cellulose sodium [CMC-Na]/saline), fluoxetine (Flu, 10 mg/kg), EFR (1.5 g of crude drug per kilogram), EFB (1.5 g of crude drug per kilogram), EMR (1.5 g of crude drug per kilogram), PFR (1.5 g crude drug per kilogram), PFB (1.5 g of crude drug per kilogram), or PMR (1.5 g of crude drug per kilogram) for 7 days.

To examine the antidepressive actions of Rb1 involving the BDNF–TrkB–CREB signaling pathway, mice were orally administered Con (0.5% CMC-Na/saline), vehicle (Veh, 0.5% CMC-Na/saline), or Rb1 (20 mg/kg) for 21 days. Veh (0.5% CMC-Na/saline) or *N*-[2-[(hexahydro-2-oxo-1*H*-azepin-3-yl)amino]carbonyl]phenyl-benzo[*b*]thiophene-2-carboxamide (ANA-12, 0.5 mg/kg) was intraperitoneally administrated 30 min before drug treatment.

The treatments were orally administered to each group 60 min prior to the behavioral experiment, and all drug solutions were freshly prepared before use.

### Behavioral Tests

The open field test (OFT), tail suspension test (TST), forced swimming test (FST), and sucrose preference test (SPT) are commonly used methods for evaluating depression-like behavior. The OFT was performed before the other behavioral tests to exclude sedative- or motor-related abnormalities. Mice were individually placed in a corner of a wooden square box (40 cm × 60 cm × 50 cm) divided into 12 equal squares, and the number of squares crossed by all four paws was recorded for 6 min, according to the OFT procedure reported in a previous study (Salehi-Sadaghiani et al., 2012).

The TST was carried out according to the conventional method described by Steru et al. (1985). Each mouse was suspended 50 cm above the floor with their tail attached to a rope (approximately 1 cm from the tail end) for 6 min (Rodrigues et al., 2002). Mice remaining completely motionless were considered immobile, and the total immobility time was recorded during the last 4 min of observation by an independent blind observer.

The FST was conducted based on the original method by Porsolt et al. (1997), with slight modifications. Each mouse was placed in a cylindrical container (25 cm in height × 25 cm in diameter) containing 10 cm of water at 24 ± 1°C. An animal was considered immobile if it remained floating in the water in an upright position, making only very small movements to keep its head above the water. The duration of immobility was recorded during the last 4 min of the 6-min observation period by an independent blind observer.

The SPT is an important method used to assess anhedonia, the core symptom of depression. The mice were challenged with (1) adaptation to a 1% sucrose solution (w/v), whereby two bottles of a 1% sucrose solution were placed in each cage for 48 h, followed by (2) food and water deprivation for 24 h, with subsequent *ad libitum* access to a 1% sucrose solution and tap water; bottles containing equal weights of each liquid were placed in each cage. After 1 h, the volumes of sucrose solution and tap water consumed were recorded, and the sucrose preference was calculated by the following formula: SPT = sucrose intake (g)/[sucrose intake (g) + water intake (g)] × 100.

### Drug-Induced Tests

Reserpine and 5-HTP are commonly used to investigate the effects of antidepressants on adrenomimetic, dopaminomimetic, and serotonomimetic properties. The head-twitch response induced by 5-HTP was assessed in mice according to Corne et al. (1963). Thirty minutes after the last drug treatment, mice were intraperitoneally administered a single dose (200 mg/kg) of 5-HTP. Thirty minutes later, each mouse was placed in a cage, and the cumulative number of head twitches was then recorded over a 30-min period by a blind observer.

Reserpine treatment was performed according to the method described by Bourin et al. (1983). Thirty minutes after the last drug treatment, mice were intraperitoneally administered a dose of reserpine (4 mg/kg), except for those in the control group. The degree of ptosis was determined 30 min after reserpine treatment. The following scale was used to rate the degree of ptosis: 0, eye open; 1, eye one-quarter closed; 2, eye half closed; 3, eye three-quarters closed; and 4, eye completely closed (Sánchez-Mateo et al., 2007). The rectal temperature of each animal was measured by inserting a thermistor thermometer 240 min after reserpine injection. Each mouse was then placed in the center of a disk (10 cm in diameter), and the akinesia and degree of palpebral ptosis were estimated within 15 s. Mice were judged to be akinetic when presenting one or more of the following responses: (1) walking to the edge of the disk and looking over the side; (2) moving 180° in place; and (3) moving the head 90° in one direction immediately followed by a 45° movement in the opposite direction.

### Chronic Unpredictable Mild Stress Procedure

The CUMS procedure was conducted as previously described, with minor modifications (Willner et al., 1987). Mice were acclimated to the environment for 1 week before being subjected to 10 mild stressors for 5 weeks, as follows: (1) food deprivation for 12 h; (2) water deprivation for 12 h; (3) soiled cage for 24 h; (4) plantar electrical stimulation for 10 min; (5) cold water swimming at 4°C for 5 min; (6) overnight illumination for 36 h; (7) nipping of the tail for 2 min; (8) cage tilt (45°) for 12 h; (9) white noise for 12 h; and (10) LED stroboscopic stimulation for 2 h. The 10 mild stressors were randomly applied, and each was discontinuous and irregular to ensure the unpredictability of the experiment. Mice of the control group were housed in a different room to prevent interference.

### Western Blot Analysis

At the end of the CUMS protocol, the mice were decapitated using a guillotine. The hippocampal CA3 region and the PFC were immediately and carefully removed from the brain samples onto an ice-cold plate and chilled in ice-cold saline solution to remove residual blood. The hippocampal CA3 and PFC tissues were then frozen in liquid nitrogen and stored at −80°C until assay.

The hippocampal CA3 and PFC tissues of each mouse were separately homogenized in ice-cold radioimmunoprecipitation assay (RIPA) buffer and then centrifuged at 12,000 rpm at 4°C for 30 min, and the supernatant was collected. The protein concentration in the supernatant was measured using a bicinchoninic acid (BCA) protein assay kit. Total protein (20-μg aliquots) was separated by 10% sodium dodecyl sulfate polyacrylamide gel electrophoresis (SDS-PAGE) gels and electroblotted onto polyvinylidene difluoride (PVDF) membranes. The membranes were blocked with 5% (w/v) nonfat dried milk in Tris-buffered saline containing 0.1% Tween 20 (TBST) for 90 min at room temperature and then incubated overnight at 4°C with antibodies (diluted in TBST) against the following proteins: BDNF (Cat No.: NBP2-55052; 0.4 µg/ml; Novus Biologicals, Littleton, CO, USA), TrkB (Cat No.: 4603; 1:1,000), AKT (Cat No.: 4691; 1:1,000), p-AKT (Thr308) (Cat No.: 9275; 1:1,000), ERK1/2 (Cat No.: 4695; 1:1,000), p-ERK1/2 (Thr202/Tyr204) (Cat No.: 9102; 1:1,000), and CREB (Cat No.: 9197; 1:1000) (all from Cell Signaling Technology, Inc., Danvers, MA, USA) and p-TrkB (Tyr515) (Cat No.: Ab109684; 1 µg/ml) and p-CREB (Ser133) (Cat No.: Ab32096; 1:5,000) (both from Abcam, Cambridge, UK). The membranes were washed with TBST and then incubated with horseradish peroxidase (HRP)-conjugated antirabbit immunoglobulin G (IgG) antibody. Images were captured with a Nikon Digital Sight DS-Ri1 imaging system (Nikon Corporation, Tokyo, Japan), and immunoreactive bands were analyzed and quantified using ImageJ software (NIH, Bethesda, MD, USA). Protein expression was normalized to the beta-actin control.

### Statistical Analysis

All data represent the mean ± standard error of the mean (SEM). For comparisons, data were analyzed by one- or two-way analysis of variance (ANOVA) with Dunnett’s or Bonferroni’s *post hoc* test using GraphPad Prism software (version 6.01). The significance threshold was set at *p* < 0.05.

## Results

### Antidepressive Effects of the Different Medicinal Parts of Ginseng

#### Mouse Behavior in the OFT, TST, and FST

**Figure 2:**
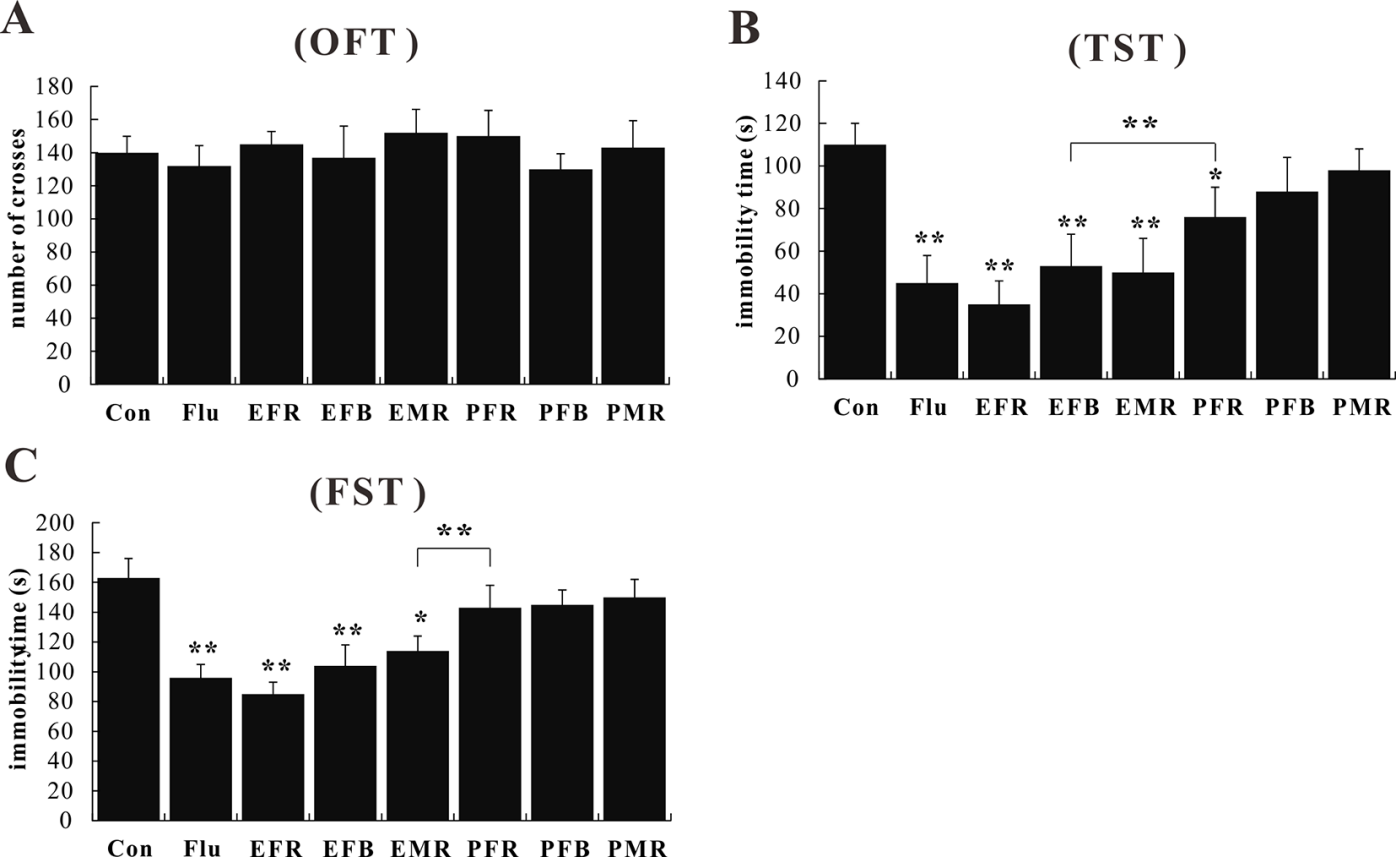
Effects of the water extract of ginseng fibrous roots (EFR), flower buds (EFB), and main roots (EMR) and the powder of ginseng fibrous roots (PFR), flower buds (PFB), and main roots (PMR) on the behavior of normal mice in the open field test (OFT) **(A)**, tail suspension test (TST) **(B)**, and forced swim test (FST) **(C)** (mean ± SEM, *n* = 10). *p < 0.05, **p < 0.01 versus the control 0.5% carboxymethyl cellulose (CMC)-Na-treated group. Comparisons were performed by one-way analysis of variance (ANOVA) with Dunnett’s test using GraphPad Prism software (version 6.01).

To assess the antidepressant-like effects of the different medicinal parts of ginseng, mice were pretreated with powders and extracts of the fibrous roots, main roots, or flower buds at the dose of 1.5 g of crude drug per kilogram. No effect on spontaneous locomotion was observed for any treatment group [*F*(7, 72) = 1.145, *p* > 0.05; **Figure 2A**]. In the TST and FST, administration of EFR, EFB, or EMR significantly attenuated the increase in immobility time [*F*(7, 72) = 4.943, *p* < 0.05; **Figures 2B, C**], and the treated mice showed significant anti-immobility behavior compared to those treated with PFR, PFB, or PMR [*F*(7, 72) = 4.332, *p* < 0.05; **Figures 2B, C**].

#### 5-HTP- and Reserpine-Induced Effects in Mice

**Figure 3:**
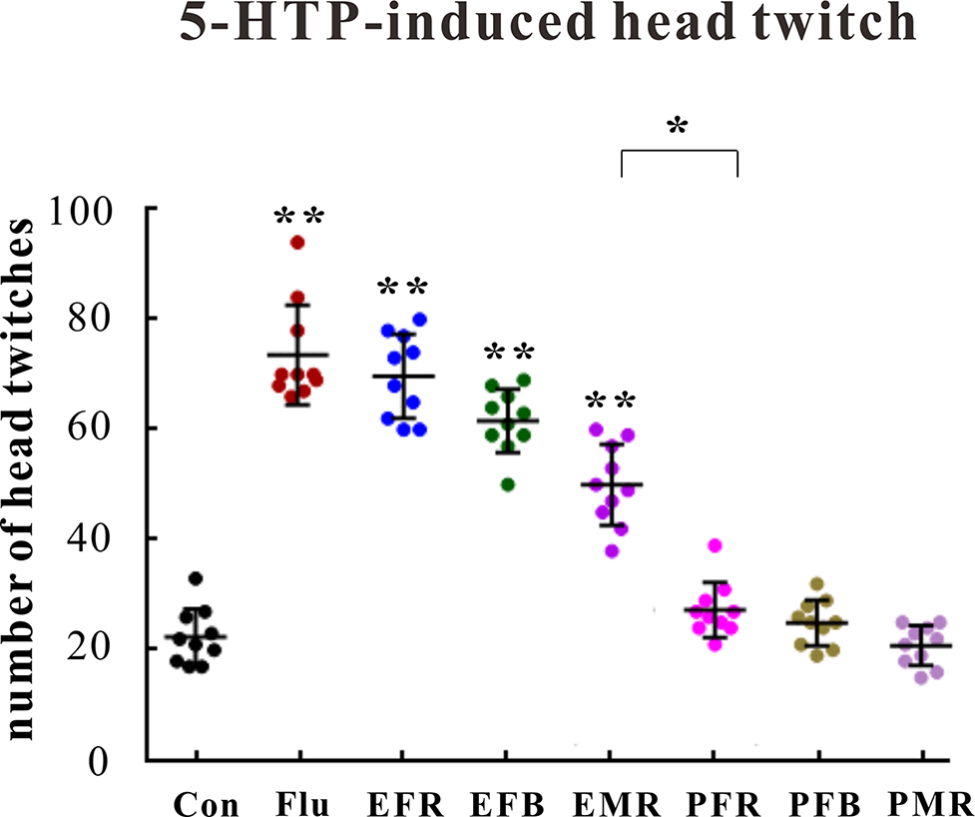
Effects of the water extract of ginseng fibrous roots (EFR), flower buds (EFB), and main roots (EMR) and the powder of ginseng fibrous roots (PFR), flower buds (PFB), and main roots (PMR) on the 5-hydroxytryptophan (5-HTP)-induced head-twitch response in mice (mean ± SEM, *n* = 10). *p < 0.05, **p < 0.01 versus the control 0.5% carboxymethyl cellulose (CMC)-Na-treated group. Comparisons were performed by one-way analysis of variance (ANOVA) with Dunnett’s test using GraphPad Prism software (version 6.01).

Control, fluoxetine (10 mg/kg), or the powders and extracts of the different ginseng medicinal parts (1.5 g of crude drug per kilogram) were administered orally 30 min after the control or 5-HTP treatment (200 mg/kg, i.p.). As shown in **Figure 3**, pretreating mice with EFR, EFB, or EMR clearly increased the number of 5-HTP-induced head twitches [*F*(7, 72) = 6.102, *p* < 0.01], whereas the number of head twitches was not substantially affected by PFR, PFB, or PMR administration [*F*(7, 72) = 1.445, *p* > 0.05].

The data presented in **Table 1** further showed that intraperitoneal reserpine injection (4 mg/kg) significantly reduced the ptosis score, locomotor activity, and rectal temperature after 240 min [*F*(7, 72) = 9.000, *p* < 0.01]. Subchronic treatment with fluoxetine at a dose of 10 mg/kg and EFR, EFB, or EMR at a dose of 1.5 g crude drug of kilogram for 7 days reversed the above effects [*F*(7, 72) = 4.000, *p* < 0.05]. Although PFR, PFB, and PMR treatments showed the opposite trends, no significant differences were recorded among the three groups [*F*(7, 72) = 1.556, *p* > 0.05].

**Table 1:** Effects of the EFR, EFB, and EMR and the PFR, PFB, and PMR on reversion of reserpine-induced ptosis, hypothermia, and locomotor activity in mice (mean ± SEM, *n* = 10).

Group	Reserpine (4 mg/kg)	Score of ptosis	Locomotor activity (%)	Rectal temperature (°C)
Control	−	0.0 ± 0.0	100.0	36.9 ± 0.4
Vehicle	+	3.6 ± 0.3^▼▼^	0.0^▼▼^	29.4 ± 0.7^▼▼^
Flu	+	1.2 ± 0.5**	90.0**	36.4 ± 0.5**
EFR	+	1.2 ± 0.3**	85.0**	36.1 ± 0.6**
EFB	+	1.4 ± 0.4**	80.0**	35.6 ± 0.4*
EMR	+	1.6 ± 0.5**	70.0*	35.4 ± 0.3*
PFR	+	2.5 ± 0.3	30.0	34.5 ± 0.9
PFB	+	2.6 ± 0.6	30.0	34.0 ± 0.5
PMR	+	2.4 ± 0.4	10.0	33.9 ± 0.9

^▼▼^p < 0.01 versus the control 0.5% carboxymethyl cellulose (CMC)-Na-treated group; *p < 0.05, **p < 0.01 versus the vehicle reserpine-treated group. Comparisons were made by one-way analysis of variance (ANOVA) with Dunnett’s test. EFR, water extract of fibrous roots; EFB, water extract of flower buds; EMR, water extract of main roots; PFR, powder of fibrous roots; PFB, powder of flower buds; PMR, powder of main roots.

### Antidepressive Effects of Rb1 in Depressive CUMS-Treated Mice

#### Behavioral Tests (OFT, SPT, TST, and FST) on CUMS-Treated Mice

**Figure 4:**
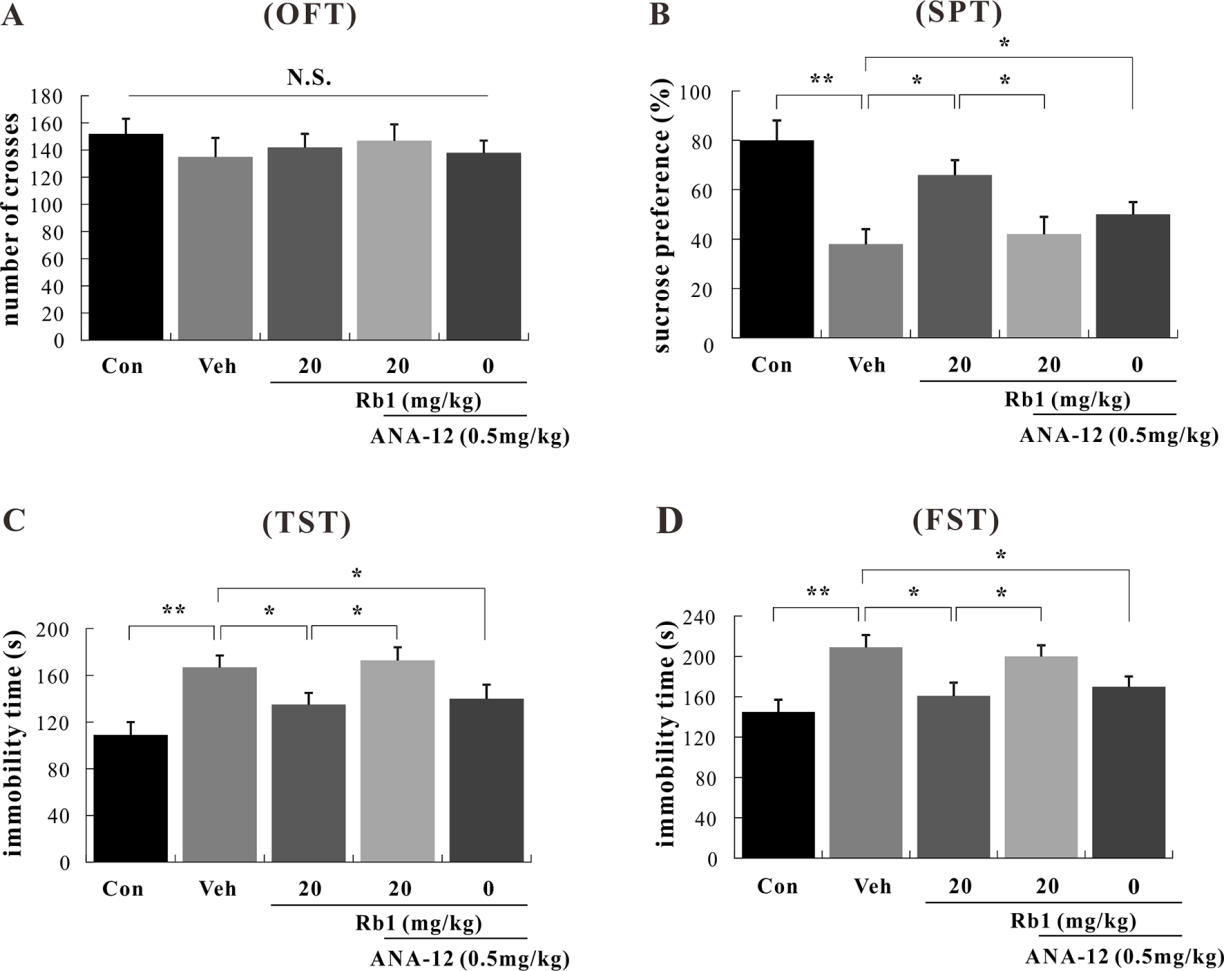
Effects of Rb1 and *N*-[2-[(hexahydro-2-oxo-1*H*-azepin-3-yl)amino]carbonyl]phenyl-benzo[*b*]thiophene-2-carboxamide (ANA-12) in the open field test (OFT) **(A)**, sucrose preference test (SPT) **(B)**, tail suspension test (TST) **(C)**, and forced swim test (FST) **(D)** in depressive chronic unpredictable mild stress (CUMS)-treated mice (mean ± SEM, *n* = 10). *p < 0.05 and **p < 0.01 were considered significant. Comparisons were performed by two-way analysis of variance (ANOVA) with Bonferroni’s *post hoc* test using GraphPad Prism software (version 6.01).

To assess the role of BDNF–TrkB in the mechanism of Rb1 action, mice were exposed to chronic and continuous mild stressors similar to those associated with human depression. The behavior of mice subjected to the OFT, SPT, TST, and FST was then assessed after the last treatment with the novel TrkB antagonist, ANA-12. In the OFT, no differences were observed between the Rb1- and ANA-12-treated groups [*F*(4, 45) = 1.009, *p* > 0.05; **Figure 4A**]. In the SPT, TST, and FST, treatment with Rb1 at a dose of 20 mg/kg significantly increased sucrose preference [*F*(4, 45) = 3.625, *p* < 0.05; **Figure 4B**] and attenuated the increase in immobility time of CUMS-exposed mice compared to the control group [*F*(4, 45) = 4.443, *p* < 0.05; **Figures 4C, D**]. However, the antidepressant-like effects of Rb1 were significantly inhibited in mice pretreated with ANA-12 at a dose of 0.5 mg/kg [*F*(4, 45) = 4.110, *p* < 0.05; **Figures 4B–D**]. Surprisingly, treatment with the TrkB antagonist ANA-12 (0.5 mg/kg, i.p.) alone had a significant effect on the behavior of mice in the SPT, TST, and FST [*F*(4, 45) = 3.761, *p* < 0.05; **Figures 4B–D**], consistent with previous reports (Yang et al., 2015; Zhang et al., 2015a; Zhang et al., 2015b).

#### Roles of BDNF–Trkb–CREB Signaling in the Hippocampal CA3 and PFC Regions of CUMS-Treated Mice

**Figure 5:**
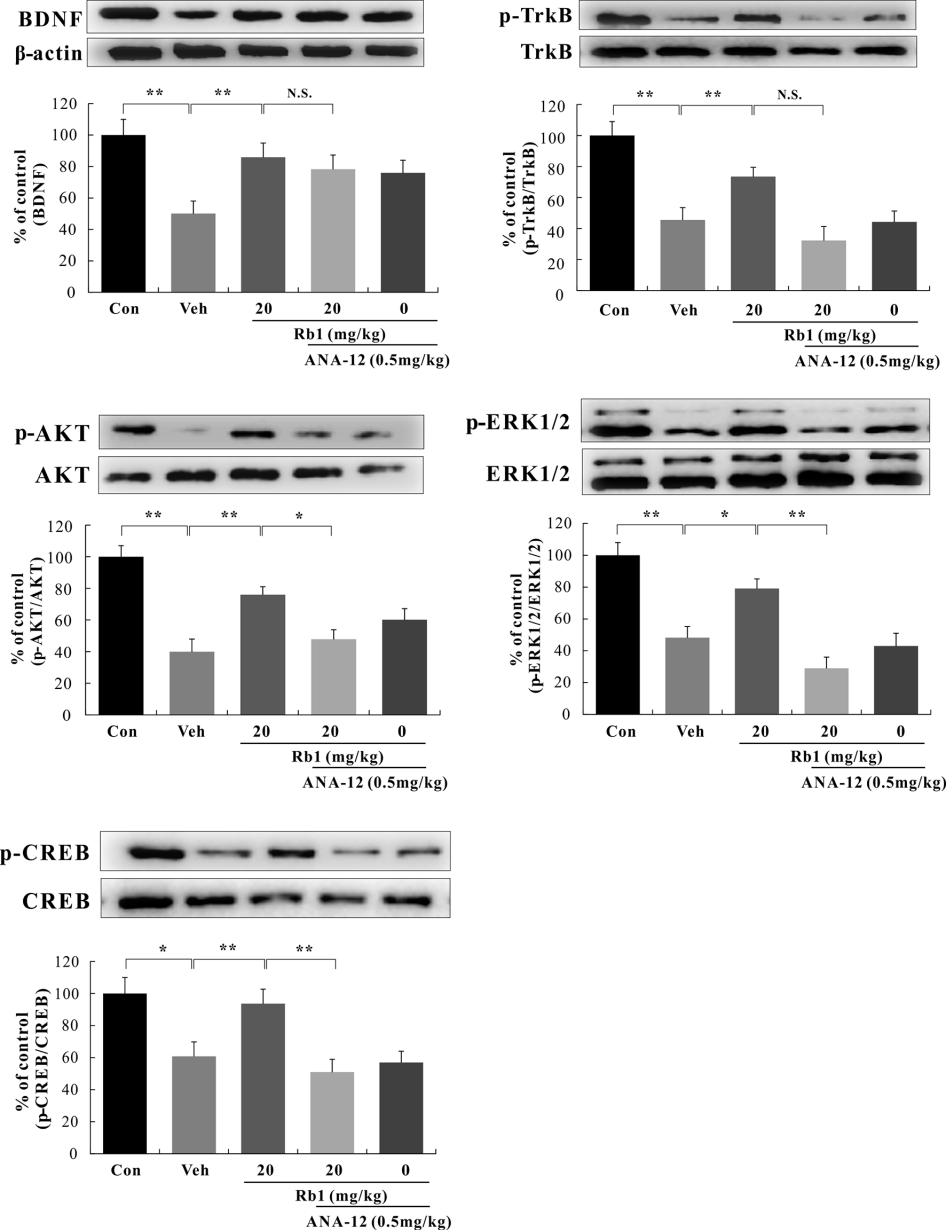
Role of *N*-[2-[(hexahydro-2-oxo-1*H*-azepin-3-yl)amino]carbonyl]phenyl]-benzo[*b*]thiophene-2-carboxamide (ANA-12) on the antidepressive action of Rb1 in the hippocampal CA3 region of chronic unpredictable mild stress (CUMS)-treated mice (mean ± SEM, *n* = 10). *p < 0.05 and **p < 0.01 were considered significant. Comparisons were performed by two-way analysis of variance (ANOVA) with Bonferroni’s *post hoc* test using GraphPad Prism software (version 6.01).

**Figure 6:**
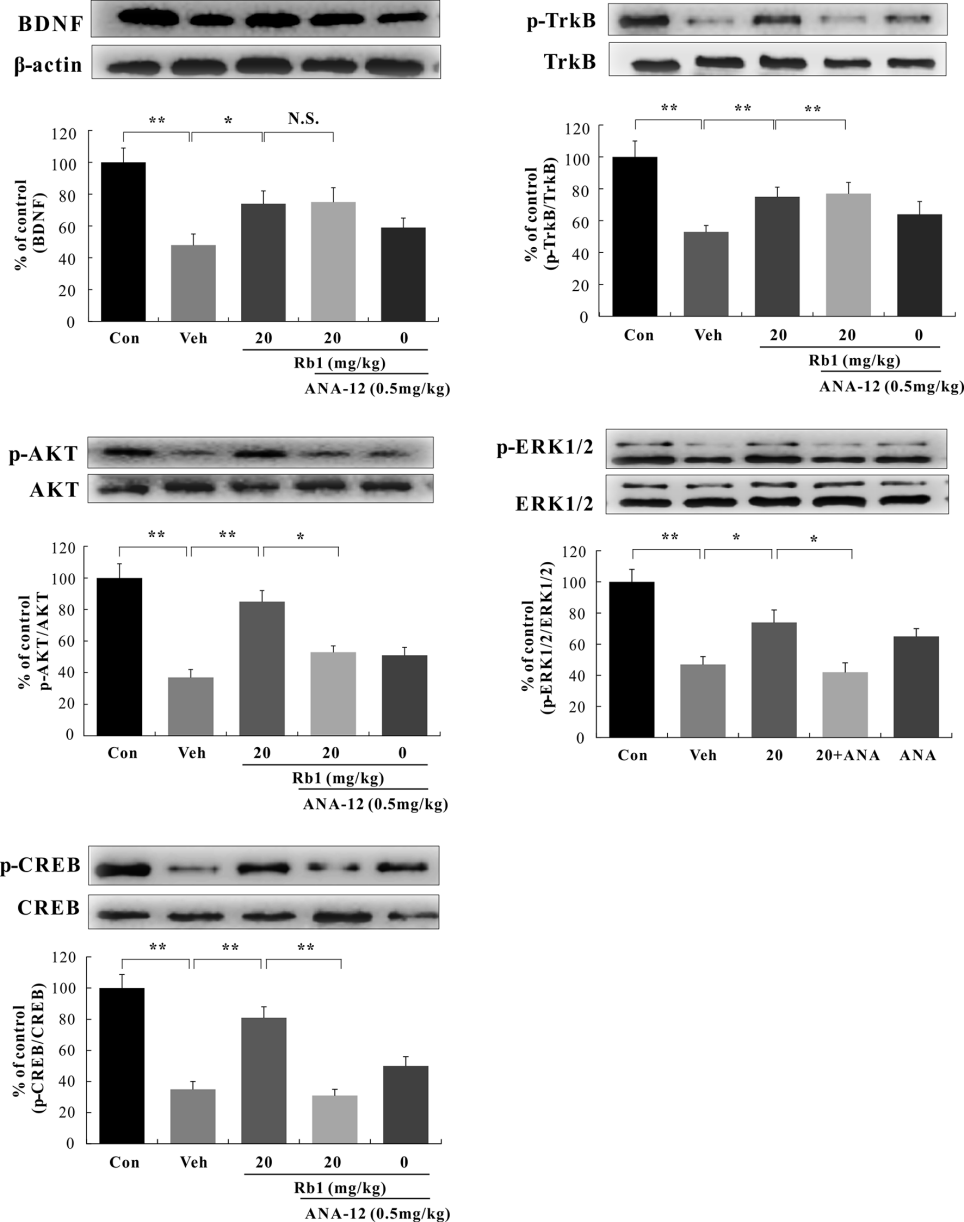
Role of N-[2-[(hexahydro-2-oxo-1H-azepin-3-yl)amino]carbonyl]phenyl]-benzo[b]thiophene-2-carboxamide (ANA-12) on the antidepressive action of Rb1 in the prefrontal cortex (PFC) of chronic unpredictable mild stress (CUMS)-treated mice (mean ± SEM, n = 10). *p < 0.05 and **p < 0.01 were considered significant. NS, not significant. Comparisons were performed by two-way analysis of variance (ANOVA) with Bonferroni’s post hoc test using GraphPad Prism software (version 6.01).

We performed a western blot analysis of BDNF–TrkB signaling in the hippocampal CA3 and PFC regions. Our data show that Rb1 treatment (20 mg/kg, p.o.) for 21 days significantly increased BDNF protein levels [*F*(4, 45) = 7.890, *p* < 0.01; **Figures 5** and **6**] and attenuated the decreased ratios of phosphorylated to unphosphorylated TrkB, AKT, ERK1/2, and CREB in the hippocampal CA3 region and PFC of CUMS-exposed mice [*F*(4, 45) = 4.772, *p* < 0.05; **Figures 5** and **6**]. Interestingly, at the level of protein expression, treatment with ANA-12 (0.5 mg/kg, i.p.) did not lead to changes in the level of BDNF in the mouse hippocampal CA3 region or PFC [*F*(4, 45) = 1.643, *p* > 0.05; **Figures 5** and **6**]. However, ANA-12 treatment completely blocked the effect of Rb1 on the expression of the TrkB receptor [*F*(4, 45) = 3.440, *p* < 0.05; **Figures 5** and **6**], indicating that TrkB has a role in the antidepressive effects of Rb1. Collectively, these results suggest that BDNF–TrkB–CREB signaling is involved in the antidepressive activity of Rb1.

## Discussion

There is currently a trend towards finding effective alternative medicines that can regulate affective disorders when administered as dietary supplements. *P. ginseng* is commonly used as a tonic and traditional Chinese dietetic herbal medicine owing to its abundance of medicinal ingredients, including ginsenosides, polysaccharides, proteins, volatile oils, amino acids, inorganic elements, peptides, vitamins, organic acids, alkaloids, fats, flavonoids, enzymes, and sterols. Ginsenosides are the main active ingredients of *P*. *ginseng*. The results of the HPLC analysis indicated that the 20 types of ginsenosides (Rg5, F2, Rb1, Rb2, Rb3, Rc, compound K, Rd, Rg3, Rh2, PPD, Rf, Rg1, Rg2, Re, F1, Rk3, Rh1, Rh4, and PPT) are differentially distributed in the roots and flower buds of 6-year-old garden ginseng.

Here, a series of experiments was carried out to evaluate the antidepressive actions of ginseng through behavioral and drug-induced tests. The TST and FST are the most widely used behavioral assays for detecting antidepressant-like activity and depression-like behavior in mice. Additionally, 5-HTP and reserpine are commonly used to evaluate the effects of antidepressants on the monoaminergic system. Our animal experiments indicated that administration of water extracts of ginseng fibrous roots, flower buds, and main roots significantly reduced immobility time in the FST and TST without affecting locomotor activity, significantly enhanced the 5-HTP-induced head-twitch response, and antagonized the action of reserpine. This evidence suggests that the ginseng-associated reduction in immobility time was linked to antidepressant-like effects. Furthermore, adrenomimetic, dopaminomimetic, and serotonomimetic properties might be involved in, and affect, the antidepressive activity of water extracts of ginseng.

Interestingly, the powders of ginseng fibrous roots, flower buds, and main roots did not elicit any antidepressant activity, which may be related to the poor bioavailability of ginsenosides after oral administration, excretion of active bile, and/or the reduction in biotransformation capacity (Liu et al., 2009). Previous *in* *vitro* work has shown that deglycosylated ginsenoside products are formed through bacterial metabolism in the intestinal lumen, and these products are more permeating and biologically active than glycosylated ginsenosides. It may be more difficult for the intestinal bacterial metabolites of ginseng powder to enter the bloodstream (Hasegawa and Uchiyama, 1998).

Rb1, a representative component of the PPDs, was abundantly present in fibrous root, main root, and flower bud extracts. We have previously demonstrated that Rb1 exerts antidepressant-like effects by regulating the levels of the neurotransmitter serotonin (5-HT), 5-hydroxyindoleacetic acid (5-HIAA), norepinephrine (NE), dopamine (DA), glutamate (Glu), and gamma-aminobutyric acid (GABA) (Wang et al., 2017; Wang et al., 2018). However, these effects may be associated with the downstream targets of BDNF (Shinoda et al., 2014; Chen et al., 2015). Most studies have indicated that BDNF and its downstream signaling pathway are closely associated with learning and memory dysfunction (Gao et al., 2018); moreover, recent reports have revealed that changes in the peripheral levels of BDNF also have an effect on normal animal behavior, such as skeletal muscle (Zhan et al., 2018). These reports indicate that BDNF is important for maintaining normal physiological activity.

BDNF, which binds with high affinity to the TrkB receptor, can modulate neurotransmission and enhance synaptic efficacy *via* a variety of both presynaptic and postsynaptic mechanisms (Soppet et al., 1991). BDNF promotes long-term potentiation in the hippocampus by enhancing the release of presynaptic neurotransmitters (Figurov et al., 1996). Furthermore, a postmortem study reported that BDNF expression was lowered in the hippocampus and PFC of suicide subjects with depression, whereas a similar reduction in BDNF was not observed in subjects treated with antidepressants (Karege et al., 2005). Peripheral, subcutaneous BDNF injection evokes antidepressant-like effects in rodents, prevents depression-induced behavior due to chronic stress, and increases cell survival in both the hippocampus and PFC (Schmidt and Duman, 2010). Various stress procedures decrease BDNF levels in the hippocampus and PFC, whereas chronic treatment with conventional antidepressants increases BDNF levels in these regions (Björkholm and Monteggia, 2016). These findings indicate that BDNF is a critical signaling molecule for nervous system development and the most extensively explored target regarding brain neuronal maturation, differentiation, and plasticity (Sousa et al., 2015; Nikoletopoulou et al., 2017), while the hippocampus and PFC are the most important sites for regulating the level of BDNF.

The binding of BDNF to TrkB can regulate at least three intracellular signaling pathways, PI3K/AKT, MAPK/ERK, and PLCg, which exert antidepressant-like effects through activation of the CREB transcription factor (Park and Poo, 2013). The AKT and ERK1/2 proteins are important downstream targets that play important roles in regulating cell proliferation, differentiation, survival, and apoptosis (Xia et al., 2002; Liu et al., 2015; Lim et al., 2016). Studies on mice with selective BDNF depletion in specific hippocampal subregions have shown that BDNF expression in the dentate gyrus (DG), but not CA1, is essential for the efficacy of antidepressants, supporting the survival and differentiation of neonatal DG granule cells. In contrast, the relationship between antidepressive activity and BDNF expression in the CA3 region remains unclear (Adachi et al., 2008).

In this study, mice were exposed to chronic and continuous mild stressors similar to those associated with human depression. We found that exposure to CUMS significantly reduced BDNF protein expression and downstream ratios of phosphorylated/nonphosphorylated TrkB, AKT, ERK1/2, and CREB in the mouse hippocampal CA3 and PFC regions, while long-term Rb1 administration (20 mg/kg, p.o.) for 21 days significantly attenuated the above-mentioned CUMS-induced effects. Surprisingly, treatment with the TrkB antagonist ANA-12 (0.5 mg/kg, i.p.) alone significantly affected the behavior of mice in the SPT, TST, and FST, consistent with previous reports (Yang et al., 2015; Zhang et al., 2015a; Zhang et al., 2015b). At the level of protein expression, although ANA-12 (0.5 mg/kg, i.p.) did not result in changes in BDNF levels in the mouse hippocampal CA3 and PFC regions, it completely blocked the effect of Rb1 on the protein expression of the TrkB receptor. Collectively, our research indicates that Rb1 exerts antidepressant-like effects in a mouse model of CUMS-induced depression by promoting BDNF signaling, thus providing new insights into the pharmacological effects of Rb1 in the treatment of depression. In addition to depression, BDNF signaling is also involved in other neurological disorders such as anxiety and epilepsy (Bahi, 2017; De Almeida et al., 2017), suggesting that Rb1 may also be beneficial for treating these diseases; however, this requires further experimental evidence. Rb1 is increasingly recognized as an important neurotrophic factor, with potential for application in ameliorating central nervous system disorders.

## Conclusion

In summary, the current study is the first to demonstrate that water extracts, but not the powders, of ginseng fibrous roots, flower buds, and main roots elicit novel antidepressive effects through their active ingredient, Rb1, likely by regulating the BDNF–TrkB–CREB signaling pathway. However, further studies are needed to investigate the crosstalk between ERK1/2 and AKT downstream of BDNF. Importantly, water-extracted ginseng fibrous roots, flower buds, and main roots have potential for use as prophylactics to prevent or minimize the recurrence of depression.

## Data Availability

All datasets generated for this study are included in the manuscript and/or **Supplementary files**.

## Ethics Statement

The animal study was reviewed and approved by the Animal Care Committee of Jilin Agricultural University (Permit No. ECLAJLAU-17005).

## Author Contributions

LZ and RZ supervised the whole experiment, GW and CL performed the practical work and completed the experiments, and YT and YW provided help during the experiments.

## Funding

This research was supported by the National Key Technology Support Program (No. 2011BAI03B010602), National Scientific and Technology Major Project (No. 2012ZX090304006), National Public Scientific Research (No. 201303111), Project of World Bank Loan of Jilin Agricultural Product Quality Safety (No. 2011-Z25), and Project Development Plan of Science and Technology of Jilin Province (No. 20130102075JC).

## Conflict of Interest Statement

The authors declare that the research was conducted in the absence of any commercial or financial relationships that could be construed as a potential conflict of interest.

## Supplementary Material

Figure S1 High-performance liquid chromatography (HPLC) chromatograms of **(A)** standard, **(B)** water extract of ginseng fibrous roots (EFR), **(C)** water extract of ginseng main roots (EMR), **(D)** water extract of ginseng flower buds (EFB), **(E)** powder of ginseng fibrous roots (PFR), **(F)** powder of ginseng main roots (PMR), and **(G)** powder of ginseng flower buds (PFB): (1) Rg1, (2) Re, (3) Rf, (4) Rb1, (5) Rk3, (6) Rc, (7) 20(R)-Rh1, (8) Rb2, (9) Rb3, (10) F1, (11) Rd, (12) Rk3, (13) F2, (14) Rh4, (15) Rg3, (16) PPT, (17) Compound K, (18) Rg5, (19) Rh2, and (20) PPD.

Figure S2
^13^C NMR spectra of Rb1.
