# Supplementary figures and images for: Rb1, the Primary Active Ingredient in Panax ginseng C.A. Meyer, Exerts Antidepressant-Like Effects via the BDNF–Trkb–CREB Pathway
Source: Front Pharmacol. 2019 Sep 13;10:1034. doi: 10.3389/fphar.2019.01034 (PMC6753202; doi:10.3389/fphar.2019.01034)

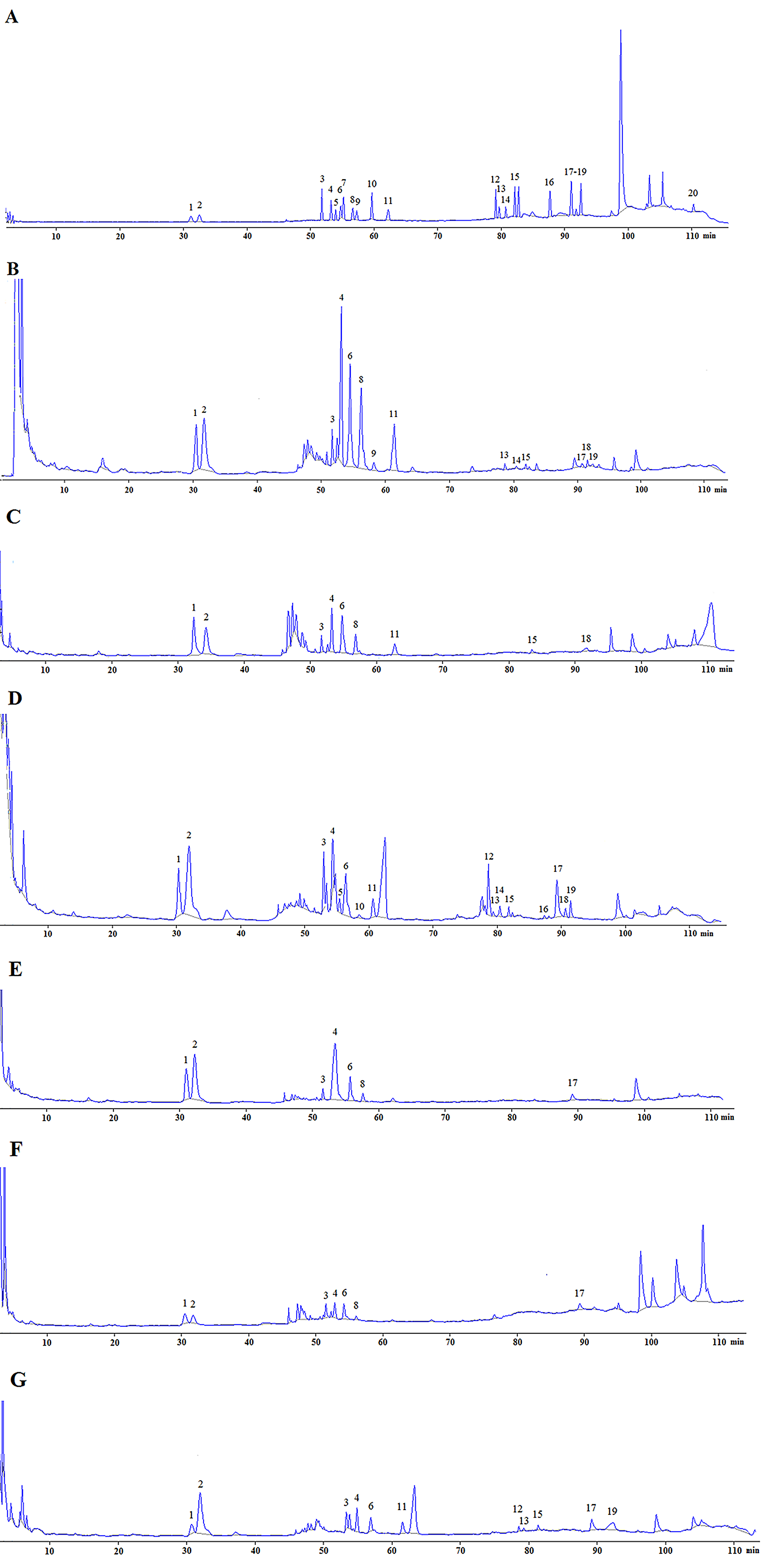

Supplement: Figure S1 — High-performance liquid chromatography (HPLC) chromatograms of (A) standard, (B) water extract of ginseng fibrous roots (EFR), (C) water extract of ginseng main roots (EMR), (D) water extract of ginseng flower buds (EFB), (E) powder of ginseng fibrous roots (PFR), (F) powder of ginseng main roots (PMR), and (G) powder of ginseng flower buds (PFB): (1) Rg1, (2) Re, (3) Rf, (4) Rb1, (5) Rk3, (6) Rc, (7) 20(R)-Rh1, (8) Rb2, (9) Rb3, (10) F1, (11) Rd, (12) Rk3, (13) F2, (14) Rh4, (15) Rg3, (16) PPT, (17) Compound K, (18) Rg5, (19) Rh2, and (20) PPD. [file Image_1.tif]

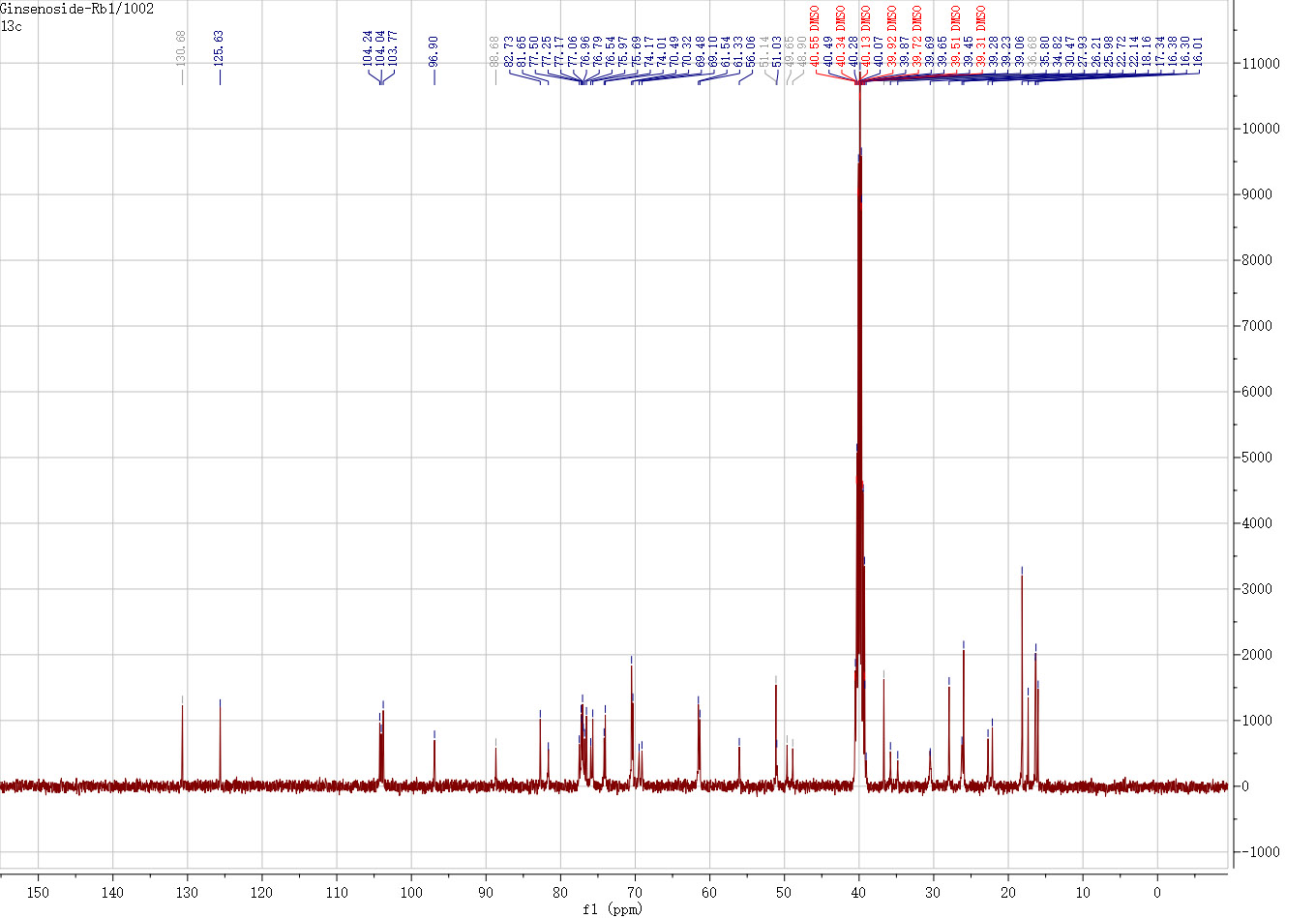

Supplement: Figure S2 — 13C NMR spectra of Rb1. [file Image_2.jpeg]
