# Supplementary material for: Rb1, the Primary Active Ingredient in Panax ginseng C.A. Meyer, Exerts Antidepressant-Like Effects via the BDNF–Trkb–CREB Pathway
Source: Front Pharmacol. 2019 Sep 13;10:1034. doi: 10.3389/fphar.2019.01034 (PMC6753202; doi:10.3389/fphar.2019.01034)
Supplement: Supplementary file 3 [file Table_1.docx]

**TABLE S1∣Content of 20 ginsenosides in different medicinal plant parts of ginseng (%)**

| Active ingredient | EFR | EMR | EFB | PFR | PMR | PFB |
| --- | --- | --- | --- | --- | --- | --- |
| Rg1 | 0.543 | 0.390 | 0.535 | 0.354 | 0.153 | 0.156 |
| Re | 0.659 | 0.295 | 1.150 | 0.510 | 0.142 | 0.491 |
| Rf | 0.221 | 0.107 | 0.522 | 0.039 | 0.036 | 0.143 |
| Rb1 | 1.292 | 0.409 | 0.784 | 0.602 | 0.108 | 0.244 |
| Rg2 | - | - | 0.089 | - | - | - |
| Rc | 0.896 | 0.396 | 0.519 | 0.201 | 0.104 | 0.145 |
| 20(R)-Rh1 | - | - | - | - | - | - |
| Rb2 | 0.783 | 0.169 | - | 0.037 | 0.009 | - |
| Rb3 | 0.036 | - | - | - | - | - |
| F1 | - | - | 0.008 | - | - | - |
| Rd | 0.587 | 0.115 | 0.154 | - | - | 0.133 |
| Rk3 | - | - | 0.348 | - | - | 0.009 |
| F2 | 0.011 | - | 0.006 | - | - | 0.003 |
| Rh4 | 0.002 | - | 0.041 | - | - | - |
| Rg3 | 0.007 | 0.004 | 0.029 | - | - | 0.011 |
| PPT | - | - | 0.007 | - | - | - |
| Compound K | 0.008 | - | 0.350 | 0.021 | 0.020 | 0.135 |
| Rg5 | 0.012 | 0.007 | 0.038 | - | - | - |
| Rh2 | 0.001 | - | 0.075 | - | - | 0.131 |
| PPD | - | - | - | - | - | - |
| Total | 5.058 | 1.892 | 4.755 | 1.764 | 0.572 | 1.601 |

Note: “-” was not detected.
