# Supplementary material for: Rb1, the Primary Active Ingredient in Panax ginseng C.A. Meyer, Exerts Antidepressant-Like Effects via the BDNF–Trkb–CREB Pathway
Source: Front Pharmacol. 2019 Sep 13;10:1034. doi: 10.3389/fphar.2019.01034 (PMC6753202; doi:10.3389/fphar.2019.01034)
Supplement: Supplementary file 4 [file Table_2.docx]

**TABLE S2∣^13^C-NMR data for Rb1 (100 MHz, DMSO-d6)**

| Carbon no. | Rb1 | Carbon no. | Rb1 |
| --- | --- | --- | --- |
| 1 | 39.23 | 28 | 27.93 |
| 2 | 26.21 | 29 | 16.38 |
| 3 | 88.68 | 30 | 17.34 |
| 4 | 40.07 | 3-O-glc-1’ | 103.77 |
| 5 | 56.06 | 2’ | 82.73 |
| 6 | 18.16 | 3’ | 76.54 |
| 7 | 34.83 | 4’ | 70.32 |
| 8 | 39.72 | 5’ | 76.79 |
| 9 | 49.65 | 6’ | 61.54 |
| 10 | 36.68 | 2’-O-glc-1’’ | 104.24 |
| 11 | 30.47 | 2’’ | 75.69 |
| 12 | 69.48 | 3’’ | 76.96 |
| 13 | 48.90 | 4’’ | 70.49 |
| 14 | 51.14 | 5’’ | 77.06 |
| 15 | 30.47 | 6’’ | 61.33 |
| 16 | 26.21 | 20-O-glc-1’’’ | 96.90 |
| 17 | 51.03 | 2’’’ | 74.01 |
| 18 | 16.01 | 3’’’ | 77.17 |
| 19 | 16.30 | 4’’’ | 70.49 |
| 20 | 81.65 | 5’’’ | 75.97 |
| 21 | 22.14 | 6’’’ | 69.10 |
| 22 | 35.80 | 6’’’-O-sugar-1’’’’ | 104.04 |
| 23 | 22.72 | 2’’’’ | 74.17 |
| 24 | 125.63 | 3’’’’ | 77.25 |
| 25 | 130.68 | 4’’’’ | 70.49 |
| 26 | 25.98 | 5’’’’ | 77.50 |
| 27 | 18.16 | 6’’’’ | 61.54 |
